# Closing the Knowledge Divide: A New Approach to Dementia‐Specific Education for Nursing Assistants

**DOI:** 10.1002/alz70858_105780

**Published:** 2025-12-25

**Authors:** Sara J Crance, Diane Nunez

**Affiliations:** ^1^ Arizona State University, Phoenix, AZ, USA; ^2^ Hospice of the Valley, Phoenix, AZ, USA

## Abstract

The prevalence of dementia is rising worldwide, with global projections exceeding 152.7 million individuals within the next 25 years. Nursing assistants (NAs) play a crucial role in providing direct care for individuals with dementia across all healthcare settings. However, dementia‐specific education is not required in most states’ NA program curricula. To address this gap, a one‐day dementia‐specific training was provided to NA program instructors. The training included dementia basics, person‐centered care, simulations, teaching techniques, and two one‐hour lessons for classroom implementation. This project aimed to evaluate the impact of incorporating this Train‐the‐Trainer dementia education program into the NA curriculum on students’ confidence in dementia care, attitudes toward dementia, and clinical practice.

Pre‐evaluation data was collected from 89 NA students in four cohorts across three Arizona community college programs. Participants were primarily female (83.15%) and ranged in age from 18 to 54 (mean = 27.99). Of these, 32.58% identified as White, 29.21% as Hispanic or Latino, and 13.48% as African American/Black, with 58.43% reporting some college education. Additionally, 44.94% of participants knew someone with dementia, and 19.10% had prior experience caring for individuals with dementia.

The Confidence in Dementia Scale (CODE) and Dementia Attitudes Scale (DAS) measured students’ confidence and attitudes before and after the education. Confidence scores significantly improved from 25.88±8.30 to 35.96±5.76 (t=‐11.90, *p* < .001), while attitudes increased from 102.77±14.50 to 112.96±15.05 (t=‐6.65, *p* < .001). Open‐ended responses revealed themes of increased confidence, patience, empathy, and understanding. NA instructors reported using the training in lectures and labs but identified time constraints and limited resources as barriers to providing dementia‐specific education.

The findings demonstrate that dementia‐specific education enhances NA students’ confidence and attitudes, fostering greater empathy and understanding in dementia care. Integrating this training into NA programs addresses critical gaps in dementia education, equipping future NAs to deliver high‐quality, person‐centered care.

